# Differences in run-up, take-off, and flight characteristics: successful vs. unsuccessful high jump attempts at the IAAF world championships

**DOI:** 10.3389/fspor.2024.1352725

**Published:** 2024-02-06

**Authors:** Gareth Nicholson, Gaspar Epro, Stéphane Merlino, Josh Walker, Athanassios Bissas

**Affiliations:** ^1^ Carnegie School of Sport, Leeds Beckett University, Leeds, United Kingdom; ^2^ Sport and Exercise Science Research Centre, School of Applied Sciences, London South Bank University, London, United Kingdom; ^3^ International Relations & Development Department, World Athletics, Monte Carlo, Monaco; ^4^ School of Education and Applied Sciences, University of Gloucestershire, Gloucester, United Kingdom

**Keywords:** kinematics, biomechanics, videography, coaching, competition

## Abstract

Studies previously conducted on high jump have yielded important information regarding successful performance. However, analyses in competitive scenarios have often disregarded athletes’ unsuccessful attempts. This study aimed to investigate the biomechanical differences between successful and unsuccessful jumps during competition. High-speed video footage (200 Hz) was obtained from 11 athletes during the 2018 Men's World Athletics Indoor Championship Final. From each athlete, one successful (SU) and one unsuccessful (UN) jump at the same bar height were included in the analysis, leaving seven athletes in total. Following whole-body 3D manual digitization, several temporal and kinematic variables were calculated for the run-up, take-off, and flight phases of each jump. During SU jumps, athletes raised the center of mass to a greater extent (*p *< 0.01) from take-off. Touchdown in SU jumps was characterized by a faster anteroposterior velocity (*p *< 0.05), lower backward lean (*p *< 0.05), and changes in joint angles for the stance and trail limbs (*p *< 0.05). Athletes also shortened the final contact time during SU jumps (*p *< 0.01) after producing a longer flight time in the final step of the run-up (*p *< 0.05). Elite-level high jumpers undertake a series of adjustments to successfully clear the bar after UN jumps. These adjustments reinforce the importance of the run-up in setting the foundations for take-off and bar clearance. Furthermore, the findings demonstrate the need for coaches to be mindful of the adjustments required in stance and trail limbs when looking to optimize feedback to athletes during training and competition.

## 1 Introduction

The main objective of the high jump event is for the jumper to raise their center of mass (CM) to a maximum height while crossing the bar. As the high jump event has been contested since the first modern Olympiad, it is not surprising that a great number of techniques have been adopted in order to achieve this (1). Although almost all modern high jumpers utilize the Fosbury Flop technique (2), this technique still allows a wide range of technical variations compared to other jumps. There are a wealth of biomechanical data available for the high jump, which describes the run-up run, take-off (TO), and flight phases (3–7); this forms the basis of current technical models used by coaches to develop strategies to achieve maximum technical efficiency.

Analyses of the high jump have mainly been conducted during international competitions to describe the performances of the best athletes. The advantage of conducting analyses during official competitions is the increased ecological validity, both in terms of the physical environment as well as the motivation of athletes and pressure of competitive situations. Biomechanical analyses of the high jump have highlighted key performance determinants that provide key reference points for coaches to build their technical models and can be used by athletic federations to build their monitoring and talent identification programs. Several authors have stated that the take-off is the most important phase of the high jump (5, 8, 9) with the peak height of the CM during flight being heavily dependent on the height and vertical velocity of the CM at take-off. Although a great deal of attention has rightly been focused on the critical determinants of these two take-off parameters (10–12), a successful bar clearance based on an optimal supine layout position at the peak of the jump is a function of several run-up, take-off, and flight characteristics. Indeed, it has recently been demonstrated (13) that modern athletes make use of hip–shoulder rotations during the take-off phase to generate long axis rotation and enable the athlete to move from a forward-facing take-off position to a supine bar clearance position. The characteristics of the high jump undoubtedly necessitate high levels of reactive strength, lower-body stiffness, and flexibility (14, 15), but it is the technical complexity of the event which dictates that successful jumps offer a limited window for errors in execution.

Athletes in jump events are known to make small modifications in their movement patterns as they adjust their technique in response to external feedback to ensure a consistent take-off point (16). These adjustments might provide useful compensatory changes ensuring a consistent performance outcome but can also introduce a level of dysfunctional variability that may lead to unsuccessful jumps (17). Analyses of unsuccessful high jumps could yield important information about the factors contributing to suboptimal performance. While successful (SU) and unsuccessful (UN) jump comparisons have been undertaken for other events (18), key performance parameters in the high jump have mainly been identified by comparing SU jumps of different heights (6, 9, 19). As such, limited information exists on the magnitude and type of changes made by athletes to bring about successful bar clearance following an unsuccessful jump. Considering that modern, world-class high jumpers display great technical variation in jump styles (6, 7), some degree of inter-individual variability in the adjustments made following UN jumps may be expected (3, 20). Nevertheless, it would be useful to understand the extent to which modern, world-class high jumpers display common and significant differences between SU and UN jumps. In particular, information on the changes that occur in the temporal and kinematic characteristics of the run-up, take-off, and flight would be useful to inform coaches in the design of corrective strategies relating to bar clearance.

At present, there is limited information that compares the differences between SU and UN high jumps during competition, particularly in world-class performers. This information is needed to better understand the adjustments made by world-class jumpers to achieve bar clearance after UN jumps. There is limited research across all standards of high jump athletes; however, research into the highest level of performers can be used by coaches as models of excellence to inform their technical and conditioning strategies. Thus, the aim of this study was to investigate the kinematic differences between SU and UN jumps in the men's high jump final of the 2018 World Indoor Athletics Championships. The purpose of the study was to provide coaches with information on the most important factors determining successful bar clearance, which can be used to optimize feedback to athletes between jumps and thus inform corrective strategies in technique.

## 2 Methods

### 2.1 Participants

Data were collected as part of the Birmingham 2018 IAAF World Indoor Championships Biomechanics Projects. The collection and use of the data was approved by World Athletics (the owner and controller of the data) and locally approved through institutional research ethics procedures (Leeds Beckett University Ethics Sub-committee; application reference: 61250). Jumps from seven male world-class high jumpers were analyzed (body mass: 76.8 ± 7.2 kg; body height: 1.94 ± 0.04 m; personal best: 2.32 ± 0.03 m). To address the aim of the study, only athletes (seven of the 11 finalists) performing an SU and a UN attempt at the same bar height were analyzed. This height (2.25 ± 0.06 m) was not the same for all athletes as it was dictated by individual performances.

### 2.2 Protocol

Four high-speed cameras (Sony PXW-FS5) operating at 200 Hz (shutter speed: 1/1,250 s; ISO: 2,000–4,000; FHD: 1,920 × 1,080 pixels; progressive scan) were used to record the high jump action during the men's final competition commencing three steps before take-off and ending when the athlete had landed. A standardized calibration procedure was conducted before and after the competition using a rigid cuboid calibration frame measuring 3.044 m^3^ and comprising 24 reference points (7). The recorded video ﬁles were imported into a 3D movement analysis software (SIMI Motion version 9.2.2, Simi Reality Motion Systems GmbH, Germany) with one SU attempt and one UN attempt for each athlete then being manually digitized by a single experienced operator. An event synchronization technique (synchronization of four critical instants) was applied through SIMI Motion to synchronize the two-dimensional coordinates from each camera involved in the recording. The digitizing involved a continuous whole-body analysis throughout the take-off and flight phases of each jump. A 17-point whole-body model was digitized beginning three steps from the final take-off position and ending following complete bar clearance by each athlete. In accordance with de Leva (21), the 17 digitized points were the center of the head and bilaterally shoulder, elbow, wrist, metacarpophalangeal, hip, knee, ankle, and metatarsophalangeal (MTP) joint center. Each file was first digitized frame by frame, and upon completion, adjustments were made as necessary using the points over frame method (22). The reliability of the digitizing process showed minimal total errors (intraclass correlation coefficient >0.97) when it was repeated for specific variables for five randomly selected athletes with an intervening period of 48 h. The Direct Linear Transformation (DLT) algorithm (23) was used to reconstruct the real-world 3D coordinates from individual camera's x and y image coordinates. de Leva’s (21) body segment parameter models were used to obtain data for the whole-body CM and for key body segments. A recursive second-order, low-pass Butterworth digital ﬁlter (zero phase lag) was employed to filter the outcome variable with a 7–15 Hz cut-off frequency range determined through residual analysis (Winter, 2009).

Following data processing, a number of key (6, 7, 13) kinematic variables were computed to characterize the run-up, take-off, and flight phases of each athlete's jump (Table 1). The contact and flight phases of the final three steps of the run-up were categorized in an incremental order with 1 and 2 being the take-off and penultimate contacts, respectively. Athlete's body heights were obtained (24) and used to scale a number of linear displacement (horizontal and vertical) variables to account for differences in stature. In line with previous analyses (13), shoulder–hip separation angles were calculated using a custom-written Matlab script (version R2021b, MathWorks, Inc., Natick, MA, USA).

**Table 1:** Definitions for all variables analyzed.

Variable	Definition
Hip–shoulder separation angle	The angle between a vector joining the right and left hips and a vector connecting the right and left shoulders
CM height	The vertical height of the CM at TD and TO during the final foot contact (take-off)
Take-off distance	The foot–tip distance (anteroposterior) from the bar at take-off
CM–foot distance	The horizontal distance (resultant) between the CM and stance foot
Step-to-bar angle	The angle between respective foot contacts relative to the bar
Step length	The displacement between toe-off of consecutive foot contacts
Contact time	The time spent in contact with the ground during foot contact
FT:CT ratio	The ratio of flight time 1 to contact time at take-off
Horizontal CM velocity	The horizontal velocity (resultant of anteroposterior and mediolateral components) of the CM
Vertical CM velocity	The vertical velocity of the CM at different time instants
ΔCM velocity	The percentage change in CM velocity between the TD and TO of the take-off phase
Take-off angle	The angle of the CM relative to the horizontal at the instant of take-off
Knee joint angle	The angle of the thigh relative to the shank (180° = full extension)
Ankle joint angle	The angle of the shank relative to the foot (180° = full plantar flexion)
Peak CM height	The maximum vertical height of the CM during bar clearance
Time to peak height	The time period between TO and peak CM height (during bar clearance)
Peak CM distance	The anteroposterior distance of the CM from the bar at the instant of peak CM height (minus values indicate peak CM beyond the bar)
Peak pelvis height	The maximum vertical height of the pelvis during bar clearance
Peak pelvis location	The anteroposterior distance of the pelvis from the bar at the instant of peak pelvis height (minus values indicate peak pelvis height beyond the bar)
CM raise	The vertical distance between peak CM height (during flight) and CM height at take-off
CM flight distance	The horizontal distance (resultant) between the CM at TO (during the take-off phase) and the CM at peak height (during the flight phase)

### 2.3 Statistics

Results are reported as means ± standard deviations (SD). All statistical analyses were carried out using SPSS Statistics 26 (IBM SPSS, Inc., Chicago, IL, USA) and distribution parameters were used to check the appropriateness of parametric tests. Paired samples *t*-tests were then used to quantify the differences between SU and UN jumps; significance was set at *p* < 0.05 (25). Cohen's *d* (26) was used as an effect size to determine the magnitude of differences between successful and unsuccessful jumps with interpretation thresholds of 0.2 (small), 0.5 (medium), 0.8 (large), 1.2 (very large), and 2.0 (huge).

## 3 Results

Concerning the velocity-related variables (Table 2), a significant difference was observed in anteroposterior CM velocity at touchdown (TD) (*p *< 0.05, *d *= 0.015) with the SU jumps demonstrating a 6.3 ± 5.1% faster velocity than the UN jumps. No other differences were observed for any of the velocity-related variables. For the spatiotemporal variables (Table 2), a significant difference was observed in ground contact time (CT) at take-off (*p* < 0.01, *d* = 0.835) and at contact 4 (*p* < 0.05, *d* = 0.847) during the run-up. Specifically, the UN attempts were characterized by a 6.2 ± 3.2% and 5.1 ± 3.3% longer contact time than the SU attempts at contact 4 (initiation of the third step before take-off) during the run-up and at take-off, respectively. The longer contact time at take-off in the UN attempts was preceded by a significantly shorter flight time (FT) (18.8 ± 15.4%, *p *< 0.05, *d *= 0.847) as well as a significantly lower FT:CT ratio in the final (take-off) step (22.5 ± 16.1%, *p *< 0.05, *d *= 0.917). The longer contact time at take-off was also characterized by a longer duration of knee extension (19.4 ± 29.2%, *d *= 0.826) during UN attempts, although this large effect did not reach statistical significance.

**Table 2:** Kinematic and spatiotemporal characteristics of the run-up and take-off for SU and UN attempts.

Variable		Mean ± SD	% diff (Cohen's *d)*	P1 % diff	P2 % diff	P3 % diff	P4 % diff	P5 % diff	P6 % diff	P7 % diff
Step to bar angle (°)	SU	28.70 ± 8.20	−6.39 ± 9.36% (*d* = 0.184)	−1.18	−9.72	−8.56	−16.46	−16.60	9.61	−1.79
	UN	27.10 ± 9.18								
Step 3 length (m)	SU	2.02 ± 0.24	0.01 ± 3.86% (*d *= 0.015)	−5.14	5.49	1.39	0.35	3.72	−3.18	−2.58
	UN	2.02 ± 0.22								
Step 2 length (m)	SU	2.18 ± 0.22	−1.56 ± 4.11% (*d *= 0.165)	−1.00	5.23	−4.58	−2.64	−4.71	−5.76	2.53
	UN	2.14 ± 0.21								
Step 1 length (m)	SU	1.97 ± 0.13	−2.53 ± 3.16% (*d *= 0.341)	−5.31	3.42	−3.42	−2.76	−1.06	−2.36	−6.24
	UN	1.92 ± 0.16								
Take-off distance (m)	SU	1.13 ± 0.17	6.39 ± 8.11% (*d *= 0.410)	13.77	12.22	−0.55	7.83	14.80	3.58	−6.94
	UN	1.20 ± 0.17^a^								
Contact 4 duration (s)	SU	0.15 ± 0.01	6.24 ± 3.21% (*d *= 0.847)	6.25	-	3.57	10.00	10.34	3.57	3.70
	UN	0.15 ± 0.01^a^								
Contact 3 duration (s)	SU	0.14 ± 0.01	−0.55 ± 8.13% (*d *= 0.060)	6.67	−3.45	−11.54	−6.90	11.54	−3.85	3.70
	UN	0.14 ± 0.01								
Contact 2 duration (s)	SU	0.14 ± 0.02	3.68 ± 8.45% (*d *= 0.223)	−2.86	7.69	17.39	7.69	−3.57	−6.67	6.06
	UN	0.15 ± 0.02								
Contact 1 duration (s)	SU	0.17 ± 0.01	5.09 ± 3.30% (*d *= 0.835)	5.88	2.94	2.70	6.25	2.86	3.23	11.76
	UN	0.18 ± 0.01^b^								
Flight 3 duration (s)	SU	0.10 ± 0.03	−8.71 ± 11.62% (*d *= 0.259)	−10.00	−14.29	−21.43	4.7	−16.67	10.00	−13.33
	UN	0.09 ± 0.03								
Flight 2 duration (s)	SU	0.13 ± 0.02	0.44 ± 5.25% (*d *= 0.000)	8.70	−4.00	4.00	−3.33	−3.03	−4.00	4.76
	UN	0.13 ± 0.02								
Flight 1 duration (s)	SU	0.05 ± 0.01	−18.83 ± 15.39% (*d* = 0.847)	−33.33	9.09	−27.27	−14.29	−14.29	−15.38	−36.36
	UN	0.04 ± 0.01^a^								
FT:CT ratio	SU	0.31 ± 0.08	−22.48 ± 16.09% (*d *= 0.917)	−37.05	5.97	−29.19	−19.33	−16.67	−18.03	−43.06
	UN	0.24 ± 0.08^a^								
Duration of knee flexion (s)	SU	0.10 ± 0.02	−3.14 ± 18.48% (*d* = 0.166)	0.00	0.00	0.00	−40.00	−10.53	14.29	14.29
	UN	0.10 ± 0.02								
Duration of knee extension (s)	SU	0.07 ± 0.01	19.37 ± 29.21% (*d *= 0.826)	14.29	8.33	9.09	83.33	18.75	−5.88	7.69
	UN	0.08 ± 0.02								
Anteroposterior CM velocity at TD (m/s)	SU	5.31 ± 0.56	6.25 ± 5.04% (*d *= 0.015)	−2.57	−14.20	−8.39	−0.58	−10.95	−4.59	−2.47
	UN	4.98 ± 0.61^a^								
Mediolateral CM velocity at TD (m/s)	SU	5.48 ± 0.73	1.42 ± 7.97% (*d *= 0.069)	15.61	0.77	−4.07	0.74	−10.16	5.26	1.78
	UN	5.53 ± 0.60								
Horizontal CM velocity at TD (m/s)	SU	7.67 ± 0.32	−1.90 ± 5.40% (*d *= 0.483)	3.64	−4.29	−6.50	3.96	−9.44	0.28	0.15
	UN	7.52 ± 0.32								
Vertical CM velocity at TD (m/s)	SU	−0.44 ± 0.27	−65.63 ± 216.30% (*d *= 0.007)	−11.23	86.76	−15.36	20.08	8.63	−550.00	1.67
	UN	−0.44 ± 0.29								
Anteroposterior CM velocity at TO (m/s)	SU	2.67 ± 0.44	6.03 ± 10.34% (*d *= 0.332)	−7.72	9.07	3.13	19.38	10.26	14.80	−6.69
	UN	2.83 ± 0.56								
Mediolateral CM velocity at TO (m/s)	SU	3.35 ± 0.38	−7.63 ± 13.51% (*d *= 0.544)	−10.33	−2.16	−15.67	3.02	−31.56	−6.60	9.87
	UN	3.09 ± 0.56								
Horizontal CM velocity at TO (m/s)	SU	4.30 ± 0.39	−1.33 ± 7.52% (*d *= 0.153)	−9.27	0.060	−5.80	8.41	−11.31	4.10	4.00
	UN	4.24 ± 0.45								
Vertical CM velocity at TO (m/s)	SU	4.62 ± 0.19	−3.30 ± 8.82% (*d *= 0.576)	−13.30	−4.79	−14.10	−4.89	−0.51	7.72	7.49
	UN	4.46 ± 0.33								
Δ horizontal CM velocity (%)	SU	−43.93 ± 4.64	−0.31 ± 8.54% (*d *= 0.042)	17.60	−6.16	−1.04	−5.93	2.58	−5.90	−3.38
	UN	−43.65 ± 5.11								
Take-off angle (°)	SU	47.11 ± 2.71	−1.26 ± 5.29% (*d *= 0.176)	−2.69	−3.40	−5.82	−8.10	7.29	2.14	1.72
	UN	46.52 ± 3.83								

The percentage differences between SU and UN are displayed for the combined sample (mean ± SD) and individual participants (P1–P7). Step 3 is the third step before take-off, and Contact 1 is take-off.

^a^ Significantly different from SU (*p *< 0.05).

^b^ Significantly different from SU (*p* < 0.01).

Regarding the postural characteristics of the take-off phase for the SU and UN attempts (Table 3), a significantly longer CM–foot distance at TD was observed for the UN attempts (4.2 ± 4.2%, *p *< 0.05, *d *= 0.803). A number of significant differences were also observed for the trail leg at TD, with the knee joint being more extended (11.2 ± 4.1%, *p *< 0.01, *d *= 1.232) and the hip joint more flexed (3.9 ± 3.8%, *p *< 0.05, *d *= 0.918) in the UN attempts. The UN attempts also demonstrated a significantly greater peak vertical thigh velocity for the trail leg at TD (9.3 ± 7.0%, *p *< 0.05, *d *= 1.099; Table 3). Differences in the take-off leg were not significant, although there was a tendency toward a difference in ankle joint angle at TD (*p *= 0.07, 4.6 ± 5.5%, *d *= 1.028). Specifically, despite not significant, the UN attempts were characterized by a seemingly more plantar-flexed ankle joint at TD but a higher degree of dorsiflexion during the take-off phase (Table 3). There were some very large individual changes in shoulder–hip separation for some athletes; however, the variables were characterized by notable individual variation in execution between athletes.

**Table 3:** Postural characteristics of the take-off phase during SU and UN attempts.

Variable		Mean ± SD	% diff (Cohen's *d)*	P1 % diff	P2 % diff	P3 % diff	P4 % diff	P5 % diff	P6 % diff	P7 % diff
CM–foot distance at TD (m)	SU	0.77 ± 0.04	4.22 ± 4.20% (*d *= 0.803)*N*	1.53	6.69	3.99	11.83	−1.48	2.83	4.17
	UN	0.80 ± 0.04^a^								
Hip angle (trail leg) TD (°)	SU	166.90 ± 4.88	−3.92 ± 3.80% (*d *= 0.918)	−1.30	−5.85	−4.83	−7.33	−6.30	−5.36	3.54
	UN	160.39 ± 8.76^a^								
Hip angle (trail leg) TO (°)	SU	94.20 ± 5.88	−0.13 ± 3.63% (*d *= 0.021)	−3.77	4.27	4.19	1.53	−1.60	−0.64	−4.88
	UN	94.07 ± 6.56								
Peak vertical thigh (trail leg) velocity (m/s)	SU	5.83 ± 0.40	9.29 ± 6.97% (*d *= 1.099)	16.83	17.83	14.36	8.15	1.65	2.80	3.41
	UN	6.37 ± 0.57^a^								
Knee angle (trail leg) TD (°)	SU	100.00 ± 8.15	11.20 ± 4.14% (*d *= 1.232)	9.85	13.10	11.87	17.70	4.40	12.72	8.76
	UN	111.19 ± 9.92^b^								
Minimum knee angle (trial leg) (°)	SU	37.37 ± 15.10	21.43 ± 30.08% (*d *= 0.412)	39.08	35.68	74.89	1.27	8.32	3.20	−12.44
	UN	43.09 ± 12.53								
Knee angle (trial leg) TO (°)	SU	73.86 ± 18.30	13.40 ± 17.03% (*d *= 0.481)	21.44	39.49	10.46	28.09	−8.23	−0.84	3.36
	UN	81.78 ± 14.36								
Peak vertical shank (trail leg) velocity (m/s)	SU	6.14 ± 0.73	6.22 ± 7.15% (*d *= 0.537)	5.23	−6.96	9.15	11.46	14.35	1.45	8.84
	UN	6.49 ± 0.56								
Hip angle (take-off leg) TD (°)	SU	148.70 ± 10.19	2.73 ± 3.93% (*d *= 0.393)	1.37	−1.43	0.72	0.84	10.72	4.14	2.74
	UN	152.63 ± 9.78								
Hip angle (take-off leg) TO (°)	SU	171.09 ± 5.43	0.61 ± 3.37% (*d *= 0.215)	5.06	−4.94	2.04	1.51	1.29	−2.84	2.15
	UN	171.99 ± 2.36								
Knee angle (take-off leg) TD (°)	SU	162.77 ± 5.69	2.74 ± 5.23% (*d *= 0.722)	1.51	−2.18	−3.62	3.26	11.47	7.22	1.50
	UN	167.05 ± 6.17								
Minimum knee angle (take-off leg) (°)	SU	140.22 ± 6.96	−2.99 ± 3.41 (*d *= 0.663)	−7.85	−0.50	0.75	−4.38	−3.82	0.84	−5.95
	UN	135.93 ± 5.94								
Knee angle (take-off leg) TO (°)	SU	170.53 ± 5.47	−0.18 ± 3.63% (*d *= 0.075)	−3.93	−2.20	2.80	−1.33	6.61	−1.04	−2.17
	UN	170.12 ± 5.27								
Ankle angle (take-off leg) TD (°)	SU	120.96 ± 4.21	4.63 ± 5.46% (*d *= 1.028)	−4.06	4.00	3.97	0.82	6.52	13.14	8.06
	UN	126.48 ± 6.33								
Minimum ankle angle (take-off leg) (°)	SU	105.05 ± 3.10	−3.75 ± 6.44% (*d *= 0.952)	−14.81	−4.47	−4.64	−7.55	−0.01	−0.23	5.45
	UN	100.99 ± 5.17								
Ankle angle (take-off leg) TO (°)	SU	132.53 ± 5.75	−0.47 ± 6.30% (*d *= 0.097)	−0.74	−5.76	−4.27	−7.50	2.73	11.08	1.16
	UN	131.82 ± 8.60								
Shoulder–hip separation TD (°)	SU	40.00 ± 12.34	−7.19 20.94 (*d *= 0.336)	1.89	−22.03	−35.29	19.23	−10.53	−21.43	17.86
	UN	36.14 ± 10.54								
Shoulder–hip separation TO (°)	SU	12.57 ± 5.03	−16.89 ± 66.69% (*d *= 0.354)	−50.00	−50.00	−93.33	19.05	90.00	37.50	−71.43
	UN	10.00 ± 8.96								
Shoulder–hip separation ROM (°)	SU	52.57 ± 10.34	−11.17 ± 24.03% (*d *= 0.561)	−3.39	−27.40	−53.06	19.15	10.42	−12.00	−11.90
	UN	46.14 ± 12.47								

The percentage differences between SU and UN are displayed for the combined sample (mean ± SD) and individual participants (P1–P7).

^a^ Significantly different from SU (*p* < 0.05).

^b^ Significantly different from SU (*p* < 0.01).

With respect to the CM and pelvis positioning during the take-off and flight phases (Table 4), no significant differences in peak CM height were detected between the attempts. From the perspective of the run-up, no differences in CM height were observed at TD but the UN attempts displayed a significantly greater CM height at TO (*p *< 0.05, *d *= 0.326). In relation to the subsequent flight phase, a significant difference in CM raise height was then observed (*p *< 0.01, *d *= 0.716) with the SU attempts raising the CM a greater vertical distance from the take-off position.

**Table 4:** Center of mass and pelvis positioning during the take-off and flight phases for SU and UN attempts.

Variable		Mean ± SD	% diff (Cohen's *d)*	P1 % diff	P2 % diff	P3 % diff	P4 % diff	P5 % diff	P6 % diff	P7 % diff
CM height at TD (m)	SU	0.90 ± 0.06	0.16 ± 2.30% (*d *= 0.019)	2.53	0.82	−1.11	−3.58	−1.28	0.81	2.93
	UN	0.90 ± 0.06								
CM height at TO (m)	SU	1.35 ± 0.06	1.32 ± 1.33% (*d *= 0.326)	1.62	1.07	1.27	0.82	2.10	−1.01	3.38
	UN	1.37 ± 0.05^a^								
Peak CM height (m)	SU	2.33 ± 0.07	−1.00 ± 1.27% (*d* = 0.342)	−0.85	0.04	−0.64	−3.47	−1.38	−1.19	0.47
	UN	2.31 ± 0.07								
Time to peak CM height (s)	SU	1.49 ± 0.13	−1.55 ± 2.08% (*d* = 0.201)	−3.72	0.71	−3.32	−3.58	−1.99	0.35	0.70
	UN	1.46 ± 0.11								
Peak CM distance (m)	SU	0.03 ± 0.16	−233.57 ± 471.97% (*d* = 0.396)	11.42	−91.57	−1,300.00	−61.94	−103.33	−18.37	−71.20
	UN	−0.02 ± 0.12								
Peak pelvis height (m)	SU	2.47 ± 0.04	−0.51 ± 1.38% (*d* = 0.242)	0.86	0.52	−1.90	−2.75	−0.61	−0.41	0.70
	UN	2.45 ± 0.06								
Peak pelvis location (m)	SU	0.12 ± 0.05	166.46 ± 598.09% (*d* = 0.738)	−84.50	−28.68	−129.84	−52.29	1,520.00	−46.28	−16.17
	UN	0.07 ± 0.07								
CM raise (m)	SU	0.97 ± 0.06	−4.17 ± 2.79% (*d* = 0.716)	−4.27	−1.28	−3.58	−9.27	−6.11	−1.49	−3.21
	UN	0.93 ± 0.06^b^								
CM flight distance (m)	SU	1.88 ± 0.16	0.45 ± 5.77% (*d* = 0.024)	0.24	−0.32	−5.22	8.50	−3.99	−4.25	8.23
	UN	1.89 ± 0.13								

The percentage differences between SU and UN are displayed for the combined sample (mean ± SD) and individual participants (P1–P7).

^a^ Significantly different from SU (*p* < 0.05).

^b^ Significantly different from SU (*p* < 0.01).

## 4 Discussion

The purpose of this study was to compare the biomechanical characteristics of SU and UN attempts during a major international competition in elite male high jumpers. Athletes displayed significant biomechanical differences in temporal and kinematic variables between SU and UN jumps identified in the run-up as well as during take-off and flight phases. During SU jumps, athletes raised the CM to a greater extent from take-off. This could be related to a reduction in backward lean at the final touchdown and changes in positioning and angular kinematics of the stance and trail limbs as well as to changes in the temporal characteristics of the run-up. For some biomechanical variables, individual variability across the athletes was displayed in the comparisons between SU and UN; this likely reflects the differences in the technical models adopted by the world-class high jumpers and the high number of degrees of freedom in a complex movement like high jump.

Given that the objective of the high jump is for the jumper to raise their CM to a maximal height to facilitate clearance of the bar, it was not surprising that differences between SU and UN jumps were apparent in the flight phase of the jumps. Although the peak height of the CM was not significantly higher in the SU jumps, the jumpers displayed a lower CM height at TO during SU jumps (1.35 vs. 1.37 m, *p *< 0.05) and subsequently managed to raise the CM a greater vertical distance up to the apex of the jump (0.97 vs. 0.93 m, *p *< 0.01, *d *= 0.716). This suggests that athletes were more able to utilize a maximal vertical propulsion during the take-off phase or that the take-off actions (including swing actions by the trail leg and arms) created more efficient rotations around the CM.

The interdependency of biomechanical variables in the high jump is well known (13) with the run-up and take-off phases laying down the foundations for the flight phase. Research has stated that the take-off is the most important phase (8) with a number of “key” variables often being the focus of attention (e.g., horizontal velocity, knee angle, CM height). Indeed, vertical velocity at take-off is known to be a major determinant for maximizing vertical CM displacement during flight (8) and a linear relationship has previously been reported (11) between run-up speed and jump height when analyzing the best jump of each athlete. The comparison of UN and SU jumps in the present study showed some large individual differences in velocity (at take-off and touchdown) but also considerable individual variability between athletes with SU jumps not always being characterized by faster run-up speeds or higher vertical take-off velocities. A notable finding, however, was the significantly higher anteroposterior velocity at TD in the SU jumps (5.31 vs. 4.98 m/s, *p *< 0.05), which was not apparent when the anteroposterior and mediolateral velocities were combined as resultant horizontal velocity. A degree of caution is required when comparing SU and UN jumps is that the observed differences may reflect the correction of “errors” that were made during UN jumps or simply that the athlete correctly performed the appropriate movement pattern during SU jumps. Whichever the scenario, it is clear that athletes ran up in the SU trials in a manner where they were traveling at a faster velocity in the direction of the landing mat at the start of the take-off phase. The faster anteroposterior velocity at TD is likely linked to the significantly shorter CM–foot distance at TD (0.77 vs. 0.80 m, *p *< 0.05, *d *= 0.803) in the SU jumps, which demonstrates a reduced backward lean, placing the athlete in a favorable position to generate lower braking impulses during the initial stages of the take-off phase. Indeed, it is known that inward and backward leaning body positions are pre-planned strategies used by jumpers to counterbalance the forward pull of inertia, control twisting/somersaulting rotation, and to increase the time spent applying force to the ground (27). It becomes clear that athletes subsequently underwent modifications in their take-off technique during SU jump, which likely resulted from their more upright body position at TD. This was apparent in a number of kinematic and temporal variables with the athletes significantly shortening their (final) ground contact time (0.169 s vs. 0.178 s, *p *< 0.01, *d *= 0.835) during SU trials, tending to land with a more dorsiflexed ankle (120.96° vs. 126.48°, *p*^ ^= 0.070, *d *= 1.028) and tending to require less time to undertake knee extension during the take-off phase (0.068 vs. 0.080 s, *p* = 0.120, *d *= 0.826).

While there has often been a focus on the positioning and action of the take-off leg within studies that have analyzed only successful jumps (9, 19), the present findings highlight that SU jumps are also characterized by significant and large changes in the trail leg during the take-off phase. Specifically, the trail leg was shown to be significantly less extended at the knee (100.00° vs. 111.19°, *p *< 0.01, *d *= 1.232) and significantly less flexed at the hip (166.90° vs. 160.39°, *p *< 0.05, *d *= 0.918) at TD in the SU attempts with the trail leg thigh segment also displaying a significantly lower peak vertical velocity during SU attempts (5.83 vs. 6.37 m/s, *p *< 0.05, *d *= 1.099). The large effect sizes demonstrate the importance of the actions performed by the trail leg, which functions as a swing element during take-off sequentially synchronized with the swinging action of the arms (28). One explanation for the need of higher vertical velocities of the trail leg thigh during take-off could be the possibly larger braking impulses in UN jumps, which would require the jumper to rely on creating higher vertical impulses with the swing elements during the take-off. The present findings, therefore, highlight that world-class high jumpers undertake simultaneous adjustments in both the take-off as well as in the trail leg to achieve bar clearance after UN jumps and that their timely coherent work is clearly of high importance for an optimal take-off execution. Given that most of the differences between SU and UN during the take-off phase were observed at TD and not TO, it seems logical that some of these differences may have been compensatory changes in response to external feedback designed to maintain consistent take-off conditions (e.g., velocity, angle). While the take-off conditions maintained a level of consistency, there were common and significant differences between SU and UN jumps, which may have represented a level of dysfunctional variability that led to a change in performance outcome (17). Although it is clear that the differences between SU and UN jumps were greatest during the take-off phase, the influence of the run-up also requires attention since its purpose is to set appropriate conditions for landing, flight, and bar clearance (29). While the athletes displayed a consistent path of run-up during SU and UN jumps, some athletes displayed larger changes in take-off distance (up to 16 cm), but these were not consistent across all athletes. The movement execution required following an UN jump is likely dependent on the technical approach adopted, and the large variation in some biomechanical variables without doubt reflects the great variation that modern world-class high jumpers display in their approach to the event (6, 7).

In terms of the common differences observable in the run-up, athletes displayed significant differences in the pattern of the final four run-up steps with the SU attempts displaying a shorter fourth (*p *< 0.05, *d *= 0.847) and final (i.e., take-off) ground contact (*p *< 0.01, *d *= 0.835). Despite the shorter final ground contact time (take-off time), the SU jumps showed a tendency for athletes to lengthen their last step prior to take-off, which resulted in a significantly longer flight time (*p *< 0.05, *d *= 0.847) and larger flight time:contact time ratio (0.24 vs. 0.31, *p *< 0.05, *d *= 0.917). These observations together with the higher anteroposterior velocity and differences in joint kinematics for both take-off and trail leg at TD as well as slightly slower longer contact time at second contact (contact prior take-off) could indicate a poorer execution of the step prior to the take-off in the UN jumps. Despite not reaching statistical significance, the ankle and knee on the take-off leg demonstrated a lower minimal angle for the UN jumps (Table 3) which alongside the longer CM–foot distance at TD may indicate larger braking impulses, which were not effectively counteracted/overcome. The differences observed in the final stages of the run-up likely influenced the joint loading at the start of take-off and the subsequent work required by the stance and trail limb in optimizing vertical propulsion. The athletes, therefore, were able to position themselves in a more beneficial body configuration for executing the take-off in the SU jump and to lift their CM to a greater vertical distance up to the apex of the jump. Thus, coaches and athletes should be mindful that differences between SU and UN jumps in world-class high jumpers manifest in the run-up and that modifications in the execution of final strides can allow the desired take-off conditions and performance outcomes. This knowledge can be used to inform analysis and corrective strategies and may be used when designing technical models that facilitate reproducible performance outcomes.

The main strength of the current study is that the data are of world-class high jump athletes (30) competing in World Championship finals; therefore, the research has high ecological validity and the results can be used by coaches as a model of excellence. One limitation is the limited sample size and the homogenous but largely individually variable nature of the sample (i.e., elite male jumpers), which may have influenced the differences that were observed. Moreover, the study is limited by the analysis of only one SU and UN trial per athlete, which is a challenge of in-competition analyses. Future data collection involving a larger, more heterogenous sample and several trials per athlete across multiple heights may help explain the factors influencing high jump success further.

## 5 Conclusions

The present findings characterize the differences between successful and unsuccessful jumps in elite-level male high jumpers. The elite athletes displayed notable individual variability between successful and unsuccessful jumps, which was likely dependent on the individual's technical approach and the implementation of this. Despite the individual variability, the athletes demonstrated several common and significant biomechanical differences between successful and unsuccessful jumps, which started in the run-up and continued into the take-off and flight phases. In converting unsuccessful trials to successful bar clearances, athletes particularly demonstrated modifications in run-up velocity, backward lean, take-off phase duration, and in positioning of the trail leg. These kinematic variables should be the focus of future studies that compare successful and unsuccessful high jump performances. Furthermore, coaches can utilize these findings to optimize feedback during training and competition when considering corrective strategies.

## Data Availability

The raw data supporting the conclusions of this article will be made available by the authors, without undue reservation.
